# NanoBRET-based detection of ligand–receptor interactions at the neuropeptide FF receptor 1

**DOI:** 10.1039/d6ra01350c

**Published:** 2026-05-20

**Authors:** Hannah Lentschat, Annette G. Beck-Sickinger

**Affiliations:** a Leipzig University, Faculty of Life Sciences, Institute of Biochemistry Bruederstr. 34 04103 Leipzig Germany abeck-sickinger@uni-leipzig.de; b LeiCeM - Leipzig Center of Metabolism, Leipzig University Leipzig Germany

## Abstract

The neuropeptide FF receptor 1 (NPFFR1) belongs to the RF-amide G protein-coupled receptor family. Even though it is a promising therapeutic target for the treatment of chronic pain, this receptor still has not been used as a drug target. A detailed understanding of its ligand binding and activation mechanisms is essential for the rational design of novel modulators. In this study, we developed a non-radioactive, nanoBRET-based ligand binding assay to investigate ligand interactions of neuropeptide FF (NPFF) and neuropeptide VF (NPVF) with the NPFFR1. Fluorescently labeled NPFF and NPVF analogs were synthesized by conjugating a 6-carboxytetramethylrhodamine fluorophore at distinct positions, while a nanoluciferase was fused to the N-terminus of the NPFFR1 to serve as the BRET donor. This approach enables quantitative measurement of ligand binding and provides insights into the relative orientation of the ligand and receptor. Distinct BRET signal profiles for NPFF and NPVF, with a smaller window for NPVF compared to NPFF when directly labeled with the fluorophore, indicate differences in the binding orientation. Furthermore, deletion of the N-terminal residues of the receptor revealed that this region is dispensable for ligand recognition and binding in the NPFFR1. The assay was confirmed for small molecule NPFFR1 ligands, such as hederagenin, offers new opportunities to explore subtype selectivity and will guide drug discovery targeting the RF-amide receptor family.

## Abstract 2

Characterization of ligand binding at the neuropeptide FF receptor 1 (NPFFR1) through a nanoBRET-based assay.

## Graphical abstract

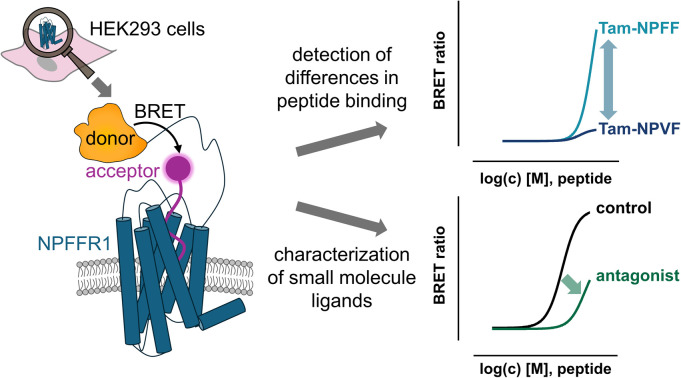

## Introduction

The neuropeptide FF receptor 1 (NPFFR1) belongs to the rhodopsin-like G protein-coupled receptors (GPCRs) and is activated by neuropeptides of the RF-amide family.^1,2^ The cell surface receptor is predominantly expressed in the central nervous system, particularly within the cerebellum, hypothalamus and spinal cord, but is also detectable at lower levels in several peripheral tissues, including adipose tissue, lymph nodes and testis.^1,3,4^ Functionally, the receptor primarily recruits the Gα_i/o_ proteins and has been connected to a broad range of physiological processes. These include nociception, regulation of blood pressure, energy homeostasis, reproduction and modulation of stress pathways.^5–9^ Previous studies highlighted the potential of selective NPFFR1 antagonists as analgesic drugs.^10–13^

The NPFFR1 shares high sequence similarity of 68% with the related receptor subtype NPFFR2.^14^ While the two endogenous ligands neuropeptide FF (NPFF) and VF (NPVF) show some cross-reactivity at both subtypes, the peptide NPFF displays higher affinity for the NPFFR2 and NPVF predominantly activates the NPFFR1.^15^ Understanding how this partial selectivity is achieved is crucial for rational drug design. Thus, despite the high physiological relevance of the NPFFR1, ligands for this receptor failed to enter clinical trials so far, mainly due to the poor CNS penetration of peptides or peptidomimetics and a lack of receptor selectivity or affinity of small molecule ligands. In 2024, the first cryo-electron microscopy (cryo-EM) structures of RF-amide receptors were published.^16–21^ In 2025, the NPFFRs bound to their endogenous ligands NPVF or NPFF and in complex with the Gα_i_ protein were resolved.^22,23^ These findings provide valuable structural insights that facilitate rational drug design, yet the functional characterization of these receptors still relies predominantly on downstream signaling assays that monitor receptor activation. Direct ligand–receptor binding assays traditionally require radio-labeled ligands, which impose practical, regulatory and technical limitations. As a modern alternative to radioligand binding assays, bioluminescence-based approaches have gained importance. In particular, bioluminescence resonance energy transfer (BRET) binding assays, using a luciferase enzyme fused to the N-terminus of the receptor together with a fluorescently labeled ligand, offer a sensitive and non-radioactive strategy to quantify ligand interactions in living cells. Such assays have already been successfully applied to multiple GPCRs, including the closely related neuropeptide Y receptor family and the β1-adrenoceptor.^24,25^ Beyond providing robust affinity measurements, these techniques can yield insights into ligand orientation and binding mode within the receptor binding pocket, thereby contributing to a deeper mechanistic understanding of receptor–ligand interactions.

To develop a non-radioactive binding assay for the NPFFR1, we synthesized a total of seven fluorescently-labeled variants of NPFF and NPVF to optimize the distance of BRET donor to acceptor and thereby increase the BRET signal. By generating an N-terminally truncated NPFFR1 variant, we significantly further increased the BRET signal and improved assay stability. Additionally, we showed that the first twenty amino acids of the receptor do not impair peptide binding. Our data confirm the different involvement and orientation of the peptide N-termini in NPFF and NPVF when bound to the NPFFR1, as suggested in the recently published cryo-EM structures.^22,23^ Validation with the NPFFR1 antagonist hederagenin confirmed the suitability of the assay for screening and characterizing novel ligands and verified the orthosteric binding mode of hederagenin at the NPFFR1.

## Experimental section

### Peptide synthesis

Peptides were synthesized by solid phase peptide synthesis using the fluorenylmethoxycarbonyl (Fmoc)/tertbutyl (*t*Bu) protection group strategy and Tentagel Resin Rink Amide (TGR RAM, IRIS Biotech GmbH) with a loading capacity of 0.19 mmol g^−1^. Automated synthesis was performed with a scale of 15 µmol per peptide and double coupling procedures using 8 eq. of amino acid (IRIS Biotech GmbH), ethyl cyanohydroxyiminoacetate (Oxyma, IRIS Biotech GmbH) and *N*,*N*-diisopropylcarbodiimide (DIC, IRIS Biotech GmbH) and a reaction time of 42 min per cycle on a SYROI synthesis robot (Multisyntech/Biotage). Fmoc cleavage was performed in two subsequent cycles by applying 40% and 20% piperidine (Sigma-Aldrich) in *N*,*N*-dimethylformamide (DMF, Biosolve) for 3 min and 10 min, respectively. 6-Carboxytetramethylrhodamine (Tam, ChemPep) was coupled on solid phase to the free peptide N-terminus or to a lysine side chain with the peptide N-terminus being Boc-protected. Coupling was performed manually using each 2 eq. Tam, hexafluorophosphate azabenzotriazole tetramethyl uronium (HATU, Novabiochem) and *N*,*N*-diisopropylethylamine (DIPEA, Roth) and incubating for 18 h under continuous shaking at room temperature.

After full cleavage with 10% TA/DODT (7 : 3) in TFA, peptides were precipitated in cold diethylether and washed. Purification was performed by preparative RP-HPLC (Aeris Peptide XB-C18 column, 100 Å, 5 µm, 250 × 21.2 mm, Phenomenex) with gradients of eluent B (0.08% trifluoroacetic acid (TFA) in acetonitrile (ACN)) in eluent A (0.1% TFA in H_2_O). Identity of the purified peptides was confirmed by matrix-assisted laser desorption/ionization time-of-flight (Microflex MALDI/TOF, Ultraflex III MALDI-TOF/TOF, Bruker Daltonics) and electron spray ionization (ESI) ion trap-orbitrap (Orbitrap Elite, Thermo Fisher Scientific) mass spectrometry. Purity of >95% was confirmed by analytical RP-HPLC on two columns (Aeris Peptide XB-C18, 100 Å, 3.6 µm, 250 × 4.6 mm and Jupiter Proteo C12, 90 Å, 4 µm, 250 × 4.6 mm (Phenomenex)).

### Plasmids & generation of Nluc-linked receptors

The pV2-hNPFFR1-eYFP plasmid with a serine at position 8.61 in comparison to arginine in the NCBI reference sequence NM_022146.4, originating from the genomic DNA of SMS-KAN cells, was used as described previously.^15,26,27^ The cDNA for the chimeric G protein Δ6Gα_qi4myr_ was obtained from E. Kostenis (Rheinische Friedrich-Wilhelms-Universität Bonn, Germany).^28,29^ The sequence encoding the Nanoluciferase (Nluc) was inserted by overlap extension PCR. The N-terminally truncated receptor variant was generated using the Q5® Site-Directed Mutagenesis Kit (New England Biolabs) according to the manufacturer's protocol. Products from the PCR or KLD reaction were transformed into heat-competent *E. coli* DH5α by a heat shock for 90 s at 42 °C and single clones were isolated on Luria–Bertani (LB, Sigma-Aldrich) medium-agarose plates containing hygromycin (130 µg mL^−1^, InvivoGen). Plasmid DNA was purified using the Wizard Plus SV Plasmid Minipreps DNA Purification System or Pureyield Plasmid Midiprep System (Promega) according to the manufacturer's protocol. All sequences were confirmed by sanger DNA sequencing (Microsynth AG).

### Cell culture

HEK293 cells (DSMZ-German Collection of Microorganisms and Cell Cultures GmbH) were cultured in DMEM/Ham's-F12 (Biowest) supplemented with 15% heat-inactivated FBS (Biochrom) and were maintained at 37 °C with 5% CO_2_ in a humidified atmosphere (standard conditions).

### Ca^2+^ assay

Ca^2+^ assays were performed with transiently transfected HEK293 cells expressing the receptor construct of interest and the chimeric G protein Δ6Gα_qi4myr_, whereby 3 µg receptor and 1 µg Δ6Gα_qi4myr_ plasmid per T25 cell culture flask were transfected using Metafectene Pro® (Biontex) according to the manufacturers' protocol. After 18 h, cells were seeded into black clear-bottom 96-well plates (Greiner, coated with 0.001% poly-d-Lys) and cultured overnight. Fluo2-AM (2.4 µM, Abcam) and Pluronic F-127 (0.5 mg mL^−1^, Sigma) in assay buffer (HBSS (Biowest), 20 mM HEPES (Sigma), 2.5 mM probenecid (Sigma), pH 7.5 at 37 °C) were added to the cells and incubated for 60 min at 37 °C. For the measurement, this solution was aspirated and replaced with assay buffer. After 20 s baseline detection, NPVF, NPFF, SQA-NPFF or a Tam-labeled peptide variant was added and the receptor-mediated Ca^2+^ response was measured for another 30 s by fluorescence readout (excitation 485 nm, emission 525 nm) in the plate reader Flexstation 3 (Molecular Devices). For analysis, the *x*-fold over basal value (RFU/baseline(avg.)) was calculated and maximal values of the Ca^2+^ response were determined. Using GraphPad Prism (version 10.6.1), the logarithmic projection of tested concentrations (*x*), was plotted against the Ca^2+^ response (*y*) and a non-linear regression (log(agonist) *vs.* response (three parameters)) was performed. Data were normalized to the control peptide (NPVF or NPFF).

### NanoBRET ligand binding assay

HEK293 cells were transiently transfected with 1.5 µg Nluc-receptor plasmid per T25 cell culture flask using Metafectene Pro® (Biontex) according to the manufacturers' protocol. After 18 h, cells were seeded into black 96-well plates (ThermoFisher, coated with 0.001% poly-d-Lys) and cultured overnight at standard conditions. Medium was replaced with assay buffer (HBSS (Biowest), 25 mM HEPES (Sigma), pH 7.4 at 37 °C), coelenterazine H and peptide were added subsequently. Luminescence and fluorescence were measured immediately after stimulation in a Spark multimode microplate reader (Tecan). BRET ratio was determined by dividing acceptor emission by the donor emission and netBRET values were determined by subtracting values of the buffer control from the BRET ratio. Using GraphPad Prism (version 10.6.1), the logarithmic projection of tested concentrations (*x*), was plotted against the netBRET ratio (*y*) and a non-linear regression (log(agonist) *vs.* response (three parameters)) was performed.

### Displacement nanoBRET assay

For displacement BRET assays, medium was replaced with different concentrations of the antagonist hederagenin (10 mM in DMSO, Biomol) or DMSO (negative control, Sigma-Aldrich) in assay buffer (HBSS (Biowest), 25 mM HEPES (Sigma), pH 7.4 at 37 °C). Subsequently, coelenterazine H and Tam-TTDS-NPVF or Tam-NPFF were added and luminescence and fluorescence were measured immediately in a Spark multimode microplate reader (Tecan). BRET ratio was determined by dividing acceptor emission by the donor emission and netBRET values were determined by subtracting values of the buffer control from the BRET ratio. Using GraphPad Prism (version 10.6.1), the logarithmic projection of tested concentrations (*x*), was plotted against the netBRET ratio (*y*) and a non-linear regression (log(agonist) *vs.* response (three parameters)) was performed. Data were normalized to the DMSO control without antagonist.

## Results & discussion

### Activity of Tam-labeled NPFF and NPVF peptides

To determine the effect of fluorophore-labeling directly or through structurally different linkers to NPFF or NPVF, the activity of these peptides was determined in a Ca^2+^ assay at the NPFFR1. The chimeric G protein Δ6Gα_qi4myr_ was used to redirect the intracellular response to the G_q_ pathway. As the 6-carboxymethylrhodamine (Tam) fluorophore is rather rigid and hydrophobic, the position of the fluorescent label in the peptide has to be optimized for minimal impact on peptide activity and binding. In addition, BRET is highly dependent on donor–acceptor-distance, possibly requiring optimization of fluorophore position in relation to the nanoluciferase (Nluc) at the receptor N-terminus.

**Fig. 1:**
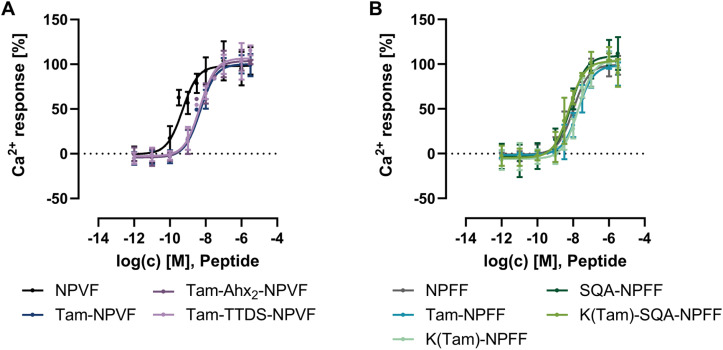
Activity of Tam-labeled NPVF (A) and NPFF (B) analogs at the NPFFR1. Ca^2+^ assays were performed in HEK293 cells transiently transfected with the NPFFR1 and the chimeric G protein Δ6Gα_qi4myr_. Data represent mean ± SEM from *n* ≥ 3 experiments.

As described, NPVF shows a lower EC_50_ value compared to NPFF^15^ with 0.5 nM and 10 nM, respectively (Fig. 1A and B and Table 1). Yet both ligands act as full agonists at the NPFFR1. Tam-labeling of the N-terminus of NPVF, either directly or through a 1,13-diamino-4,7,10-trioxatridecan-succinamic acid (TTDS) or (6-amino-hexanoic acid)_2_ (Ahx_2_) linker induced a 9- to 10-fold rightwards shift of the concentration response curve (CRC) (Fig. 1A and Table 1). While Ahx_2_ represents a flexible, hydrophobic linker that should minimize structural interference, TTDS is a hydrophilic, polyethylenglycol-like linker that can improve solubility. NPFF only loses about 2-fold in activity when directly conjugated with the Tam-fluorophore. Due to the insolubility of the Ahx_2_-linked labeled NPFF variant in a HEPES-based Ca^2+^ assay buffer, this peptide could not be tested in activity assays, as even with the addition of dimethylsulfoxide (DMSO) the peptide was not soluble. Thus, we additionally synthesized a K(Tam)-NPFF, where the fluorophore is coupled to the side chain of an additional N-terminal lysine. This peptide shows a similar loss of activity as the directly labeled Tam-NPFF. SQA-NPFF is slightly more active than NPFF with an EC_50_ value of 7.4 nM compared to 10.0 nM. When conjugated with Tam through an added N-terminal lysine side chain in K(Tam)-SQA-NPFF the peptide does not loose activity (Fig. 1B and Table 1). The loss of activity in labeled NPFF variants is likely caused by increased hydrophobicity, modification of the peptide conformation, steric hindrance or reduced solubility deriving from the Tam-fluorophore, as labeled SQA-NPFF with additional polar amino acids does not loose activity or solubility. While loss in activity for NPVF variants might also be caused, these peptides show a more pronounced EC_50_ shift compared to NPFF variants. Therefore, additional factors likely contribute. In contrast to NPFF, for NPVF an involvement of the N-terminal amino acids of the peptide has been described in high affinity receptor binding through interactions with extracellular loop 2 (ECL2).^15,22,23^ Modification with the bulky fluorophore, also through a linker, likely hampers this interaction and leads to the higher loss of activity in labeled NPVF variants. As the peptides still display 2-fold lower EC_50_ values compared to unlabeled NPFF, this interaction of the N-terminus of the peptide is not fully hindered. Overall, the fluorophore labeled peptides have EC_50_ values in the low nanomolar range and act as full agonists, thus are suitable ligands for BRET binding studies.

**Table 1:** Fluorescently-labeled NPFF and NPVF analogs. Purity was determined by RP-HPLC on two columns. EC_50_ values at the NPFFR1 were determined in Ca^2+^ assays (*n* ≥ 3) using HEK293 cells transiently transfected with the NPFFR1 and the chimeric G protein Δ6Gα_qi4myr_. The peptide Tam-Ahx_2_-NPFF was not soluble in the assay buffer, thus activity was not determined (n.d.)

Peptide	Sequence	Purity	Activity at NPFFR1	
			EC_50_	pEC_50_ ± SEM
NPVF	VPNLPQRF-NH_2_	>95%	0.5 nM	9.30 ± 0.11
Tam-NPVF	Tam-VPNLPQRF-NH_2_	>97%	4.9 nM	8.31 ± 0.08
Tam-Ahx_2_-NPVF	Tam-Ahx_2_-VPNLPQRF-NH_2_	>98%	4.4 nM	8.36 ± 0.09
Tam-TTDS-NPVF	Tam-TTDS-VPNLPQRF-NH_2_	>95%	4.8 nM	8.32 ± 0.09
NPFF	FLFQPQRF-NH_2_	>98%	10.0 nM	7.99 ± 0.07
Tam-NPFF	Tam-FLFQPQRF-NH_2_	>97%	18.4 nM	7.73 ± 0.09
Tam-Ahx_2_-NPFF	Tam-Ahx_2_-FLFQPQRF-NH_2_	>98%	n.d.	n.d.
K(Tam)-NPFF	K(Tam)-FLFQPQRF-NH_2_	>98%	19.2 nM	7.72 ± 0.08
SQA-NPFF	SQAFLFQPQRF-NH_2_	>98%	7.4 nM	8.13 ± 0.11
K(Tam)-SQA-NPFF	K(Tam)-SQAFLFQPQRF-NH_2_	>96%	5.8 nM	8.23 ± 0.09

### NanoBRET-based ligand binding assay at full-length and truncated NPFFR1

**Fig. 2:**
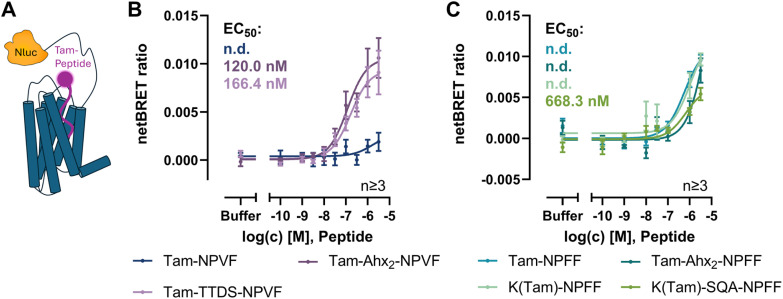
Ligand binding BRET assay at the NPFFR1 (A) with different NPVF (B) and NPFF (C) analogs. BRET assays were performed in HEK293 cells transiently transfected with the Nluc-NPFFR1-eYFP, data represent mean ± SEM of the netBRET ratio from *n* ≥ 2 experiments.

Initial ligand binding BRET assays were performed with the NPFFR1 N-terminally modified with the Nluc (Fig. 2A). The NPVF variants which were fluorescently labeled by an Ahx_2_ or a TTDS linker produced full CRCs with a maximal netBRET ratio of 0.01 and 0.009 and EC_50_ values of 120.0 nM (pEC_50_ : 6.92 ± 0.19) and 166.4 nM (pEC_50_ : 6.78 ± 0.19), respectively. Tam-NPVF, which lacks a linker structure between peptide and fluorophore, only showed a slight increase with increasing peptide concentrations and failed to produce a full CRC with the tested concentrations (Fig. 2B). As all peptides act as full agonists in activity assays, this loss in the BRET_max_ for Tam-NPVF can be attributed to a higher donor–acceptor distance or a changed orientation compared to the NPVF variants modified through a linker. Among the fluorescently labeled NPFF variants, only K(Tam)-SQA-NPFF produced a full CRC with a maximal netBRET ratio of 0.006 and an EC_50_ value of 668.3 nM (pEC_50_ : 6.18 ± 0.38) (Fig. 2C). This was also the NPFF variant displaying the highest affinity at the NPFFR1 in activity studies (Table 1 and Fig. 1B). Both Tam-NPFF without linker and K(Tam)-NPFF resulted in a concentration dependent increase in the netBRET signal up to 0.01, however, saturation was not fully reached. This indicates, that introduction of a linker in NPFF variants does not affect affinity of the peptide to the receptor, neither does it change donor–acceptor distance. This is in line with the previous findings that the N-terminal part of NPFF is rather flexible when bound to the NPFFR1, while NPVF binds in a more rigid orientation.^22^ Tam-Ahx_2_-NPFF with a longer linker showed a similar curve slightly shifted to the right. However, this peptide showed poor solubility in assay buffer, which was already observed in the activity assays. Thus, data for this peptide have to be evaluated carefully. Overall, the BRET window is rather small compared to the SEMs, suggesting further optimization of the assay.

**Fig. 3:**
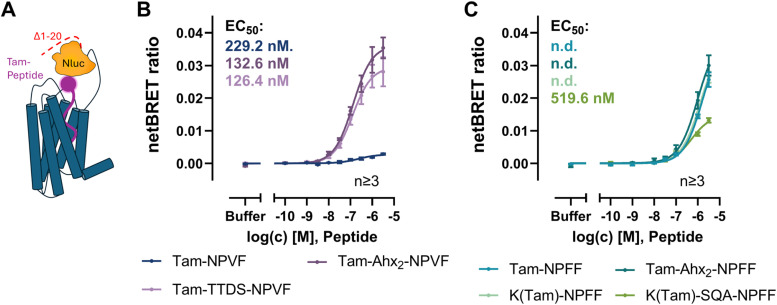
Ligand binding BRET assay with N-terminally truncated NPFFR1 (A) and different NPVF (B) and NPFF (C) analogs. BRET assays were performed in HEK293 cells transiently transfected with the Δ1-20-Nluc-NPFFR1-eYFP, data represent mean ± SEM of the netBRET ratio from *n* ≥ 3 experiments.

Previous studies described a positive effect on the BRET window by shortening the receptor N-terminus or introduction of the Nluc in ECL2.^30^ As ECL2 is involved in binding of NPVF to the NPFFR1, this loop is not suitable for insertion of the large Nluc. Thus, to improve the assay stability and increase the BRET signal, the twenty N-terminal amino acids in the NPFFR1 were deleted, resulting in the Δ1-20-Nluc-NPFFR1-eYFP receptor construct (Fig. 3A). This truncated receptor shows similar signaling as the wildtype NPFFR1 (SI Fig. S3). Again, BRET assays with Tam-Ahx_2_-NPVF and Tam-TTDS-NPVF resulted in the largest maximal netBRET ratio of 0.04 and 0.03 and full CRCs with EC_50_ values of 132.6 nM (pEC_50_ : 6.88 ± 0.08) and 126.4 nM (pEC_50_ : 6.90 ± 0.11), respectively (Fig. 3B). While the EC_50_ values are comparable to those obtained with the full-length NPFFR1, the window was significantly increased resulting in lower SEMs. A difference between full-length and truncated receptor can be seen for Tam-NPVF, where a full CRC was obtained at the Δ1-20-Nluc-NPFFR1-eYFP construct. However, this peptide showed a tenfold lower netBRET signal of 0.003 (SI Fig. S2) and a slightly higher EC_50_ value of 229.2 nM (pEC_50_ : 6.64 ± 0.17) compared to NPVF variants with linker. The lower BRET_max_ indicates a larger distance or changed orientation of fluorophore and Nluc without linker, while the reduced affinity without linker confirms that the N-terminal part of the peptide is relevant for high affinity binding. Fluorophore attachment without linker may hamper this high affinity binding due to steric hindrance or a change in polarity. Labeled NPFF variants also show an increase in netBRET ratio to 0.04 at the truncated receptor, overall showing similar results as with the full-length receptor (Fig. 3C). However, with the truncated receptor, Tam-Ahx_2_-NPFF shows similar values as the variant without linker or with linkage through a lysine side chain. Non-specific binding was excluded as reason for the increase in netBRET ratio with NPFF variants by displacing Tam-NPFF with an antagonist (SI Fig. S1). Again, only K(Tam)-SQA-NPFF shows a full CRC with a maximal netBRET of 0.01, which is significantly lower than for other NPFF variants (SI Fig. S2) and an EC_50_ value of 519.6 nM (pEC_50_ : 6.29 ± 0.10).

**Fig. 4:**
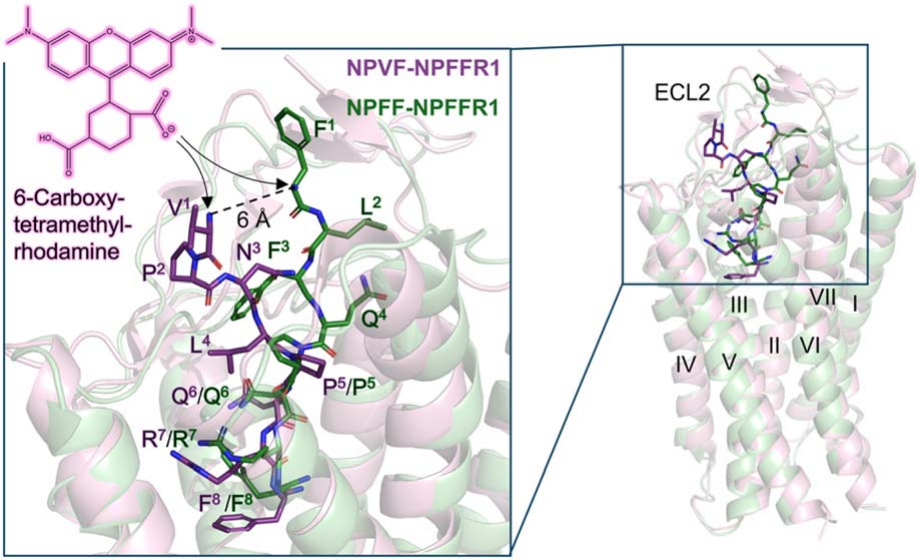
Structural differences in binding of NPFF and NPVF to the NPFFR1. Cryo-EM structures of NPFFR1 in complex with the peptide agonists (PDB 9M2F & 9M0R)^22^ confirm differential orientation of the peptide N-termini of NPVF and NPFF, resulting in different positioning of the 6-carboxytetramethylrhodamine fluorophore. Non-resolved or incompletely resolved amino acid side chains of NPFF were added in PyMOL using the mutagenesis function (F^1^, L^2^, F^3^, R^7^).

The difference of the BRET_max_ with Tam-NPVF and Tam-NPFF may indicate different binding modes of the two peptides. While the C-terminus has been described to bind deep in the binding pocket in a conserved position, the mechanism by which the N-terminus governs subtype selectivity has remained unexplored for a long time.^15^ With the cryo-EM structure available, it was shown that the N-terminal amino acids of NPVF bind in a specific position and interact with ECL2 while the corresponding ones of NPFF are not resolved in the structure.^22,23^ The structure with addition of incompletely resolved residues shows a different orientation of NPFF (Fig. 4). This indicates a distance of ∼6 Å between the N-termini of the peptides, to which the fluorophore is attached, possibly explaining the different netBRET windows. While BRET_max_ differences have to be interpreted with caution as they may reflect both, distance- and orientation-dependent effects, our results are consistent with the cryo-EM structure, indicating a different orientation of N-terminal residues of NPFF and NPVF when bound to the NPFFR1.

It has to be noted that the EC_50_ values from the binding assays are higher compared to the functional assays. However, the EC_50_ value is highly assay dependent and comparison of binding with downstream signaling assays is challenging. Furthermore, it is known that GPCR and G protein have a coupled equilibrium, with not only GPCR activation enhancing affinity for the G protein but also reciprocally.^31^ As the Ca^2+^ functional assays were performed in cells overexpressing the chimeric G protein Δ6Gα_qi4myr_ to redirect the signal from inhibition of the adenylyl cyclase to activation of protein kinase C, this might further influence the EC_50_ value. Such a difference in affinity between functional assays and BRET binding assays was also seen in previous studies at the neuropeptide Y_1_R/Y_2_R/Y_4_R.^25^ Also, radioligand binding assays at the NPFFR1 show differences to functional assays.^32^ The *K*_i_ value from this assay for NPVF is 100-fold lower compared to the pEC_50_ value from functional assays, for NPFF *K*_i_ and pEC_50_ are comparable. Unfortunately, in this study *K*_D_ values were not determined for full-length peptides, which would have enabled a better comparison between ligand binding affinity and functional potency (pEC_50_), allowing clearer interpretation of receptor occupancy-response relationships. The data from our BRET-based ligand binding assay more accurately reflect the differential binding affinities of NPVF and NPFF in agreement with functional assays and the cryo-EM structures.^22,23^

### Antagonist hederagenin reduces peptide binding at NPFFR1

**Fig. 5:**
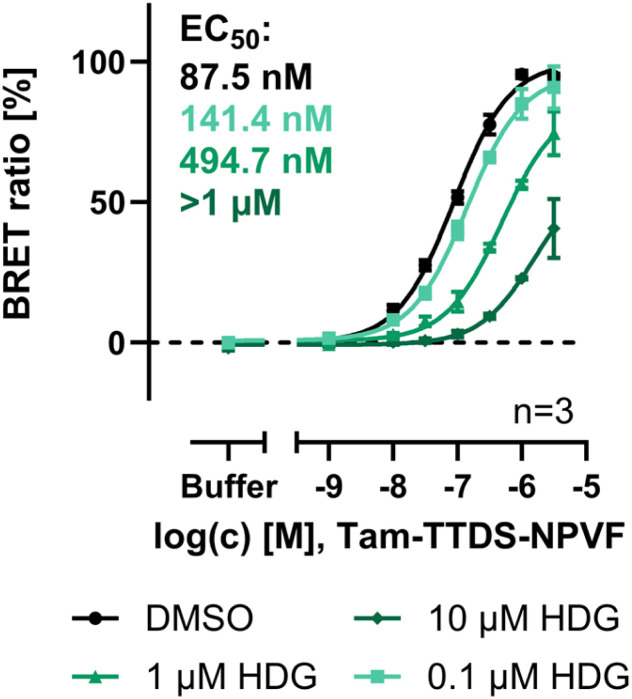
Displacement BRET assay at the N-terminally truncated Δ1-20-Nluc-NPFFR1-eYFP with Tam-TTDS-NPVF confirms antagonistic activity of hederagenin (HDG). BRET assays were performed in HEK293 cells transiently transfected with the Δ1-20-Nluc-NPFFR1-eYFP, data represent mean ± SEM of the BRET ratio normalized to the DMSO control from *n* = 3 experiments.

As the peptide Tam-TTDS-NPVF and Tam-Ahx_2_-NPVF showed good affinity and high maximal signals at the truncated NPFFR1, this set-up was chosen for the screening and characterization of new NPFFR1 ligands. As a proof of concept, the previously published selective NPFFR1 antagonist hederagenin (HDG), a triterpenoid,^27^ was tested in BRET studies with the N-terminally truncated NPFFR1 and Tam-TTDS-NPVF (Fig. 5).

Already 0.1 µM of HDG led to a slight increase in EC_50_. With increasing concentrations of HDG, the curve was further shifted to the right and the maximal signal reduced, reaching less than 50% BRET ratio with 10 µM of the compound. This confirms the effective blocking of peptide binding to the receptor and thus the orthosteric mode of action of HDG at the NPFFR1. Our data confirm, that this assay is a promising tool to screen for novel ligands targeting the NPFFR1 in living cells. Additionally, the BRET data can be used for the characterization of NPFFR1 ligands, clearing the path to exploiting the receptor's therapeutical potential.

## Conclusions

In conclusion we were able to provide a stable, non-radioactive ligand binding assay for the NPFFR1. A major advantage is the ability to investigate binding in living cells, while radioligand binding assays typically represent endpoint measurements on membranes or non-native systems and cryo-EM structures are resolved outside the cellular context.

Our findings prove that an appropriate linker between peptide and fluorophore is crucial to ensure endogenous high affinity binding without interference of the fluorophore but still adequately small flexibility to obtain a stable distance between fluorophore and Nluc, resulting in stable BRET measurements. Unfortunately, we were not able to fully compare NPFF and NPVF labeled through a linker, as Tam-Ahx_2_-NPFF shows limited solubility. Nevertheless, we identified two NPVF variants and one SQA-NPFF variant, that are suitable to perform BRET-based ligand binding assays at the truncated NPFFR1 with a large assay window and low well-to-well variations. While the BRET-based binding assay provides insights into peptide binding, the dependence of BRET_max_ not only on distance but also on fluorophore orientation necessitates comparison with structural and functional data for accurate interpretation.

Our results demonstrate that this BRET assay is not only suitable for characterizing peptide binders but also orthosteric small molecule ligands. The elevated EC_50_ values observed for NPVF variants bearing an N-terminal fluorescent label represent a limitation, as they may impede the characterization of ligands that interact predominantly with the upper region of the orthosteric binding pocket. However, such ligands would be expected to have only a minor impact, specifically on NPFF binding. Allosteric modulators that influence receptor activation without affecting ligand binding are not detectable in this assay. However, to produce robust physiological effects, such as modulation of pain perception, ligands engaging the orthosteric site of the NPFFR1 are likely required. Such ligands can be identified and characterized using the developed BRET-based binding assay, as demonstrated by the example of hederagenin.

## Author contributions

H. L.: conceptualization, data curation, formal analysis, investigation, methodology, visualization, writing – original draft, writing – review & editing. A. G. B.-S.: conceptualization, funding acquisition, project administration, resources, supervision, writing – review & editing.

## Conflicts of interest

There are no conflicts to declare.

## Supplementary Material

RA-016-D6RA01350C-s001

## Data Availability

Data supporting this article have been included as part of the supplementary information (SI). Raw data for this article, including fluorescence/luminescence measurements from Ca^2+^ and BRET assays and RP-HPLC chromatograms and mass spectra of all purified peptides, are available at Opara at https://doi.org/10.25532/OPARA-1163. Supplementary information: summary of the analytics of synthesized peptides and displacement-BRET studies with Tam-NPFF and HDG. See DOI: https://doi.org/10.1039/d6ra01350c.
