# Supplementary material for: NanoBRET-based detection of ligand–receptor interactions at the neuropeptide FF receptor 1
Source: RSC Adv. 2026 May 20;16(30):27138–45. doi: 10.1039/d6ra01350c (PMC13191738; doi:10.1039/d6ra01350c)
Supplement: RA-016-D6RA01350C-s001 [file RA-016-D6RA01350C-s001.pdf]

This journal is © The Royal Society of Chemistry 2026

Supporting Information

NanoBRET-based detection of ligand-receptor interactions at the neuropeptide FF receptor 1

Hannah Lentschat [a], Annette G. Beck-Sickinger [a]

[a] Leipzig University, Faculty of life sciences, Institute of biochemistry, Bruederstr. 34, 04103 Leipzig

Table S1: Analytics of the synthesized peptides. Experimental mass (M_{exp}) was compared to the corresponding calculated monoisotopic mass (M_{mono}). Purity of ≥ 95% was confirmed using reversed phase-high performance liquid chromatography on (a) Aeris Peptide XB-C18, 100 Å, 3.6 µm, 250 x 4,6mm (Phenomenex) and (b) Jupiter Proteo C12, 90 Å, 4 µm, 250 x 4,6mm (Phenomenex) and gradients of eluent B in A from (*) 10% to 60%, (**) 20% to 70% or (***) 30% to 80% in 40 min. All spectra and chromatograms can be accessed at Opara (https://doi.org/10.25532/OPARA-1092) Abbreviations: Ahx: 6-amino-hexanoic acid; NPFF: neuropeptide FF; NPVF: neuropeptide VF; Tam: 6-carboxytetramethylrhodamine; TTDS: 1,13-diamino-4,7,10-trioxatridecan-succinamic acid.

peptide	M_{mono} [Da]	M_{exp} $[M+H]^+$	$t_R^{(a)}$ [min]	$t_R^{(b)}$ [min]	purity [%]
NPVF (xn10)	968.56	969.54	9.9$^{(*)}$	13.8$^{(*)}$	> 95
Tam-NPVF (xw10.1)	1380.70	1381.67	12.1$^{(**)}$	16.1$^{(**)}$	> 97
Tam-Ahx$_2$-NPVF (xw10.2)	1606.87	1607.92	11.7$^{(**)}$	16.2$^{(**)}$	> 98
Tam-TTDS-NPVF (yd1)	1682.89	1683.95	19.4$^{(*)}$	12.4$^{(***)}$	> 95
NPFF (vk8+9)	1080.59	1081.59	15.5$^{(*)}$	19.1$^{(*)}$	> 98
Tam-NPFF (xw11.1)	1492.73	1493.80	15.7$^{(**)}$	19.7$^{(**)}$	> 97
Tam-Ahx$_2$-NPFF (xw11.2)	1718.90	1719.90	16.3$^{(**)}$	20.4$^{(**)}$	> 98
K(Tam)-NPFF (zw3.2)	1620.82	1621.78	12.1$^{(**)}$	19.3$^{(**)}$	> 98
SQA-NPFF (zw2)	1366.71	1367.61	8.2$^{(**)}$	15.8$^{(**)}$	> 98
K(Tam)-SQA-NPFF (zw2.2)	1906.95	1907.80	10.8$^{(**)}$	17.9$^{(**)}$	> 96

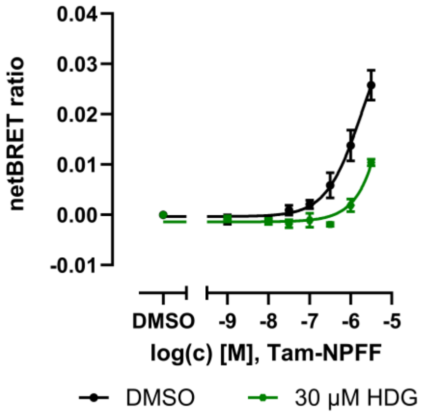

Figure S 1: Tam-NPFF at the Δ1-20-Nluc-NPFFR1 can be displaced with the selective antagonist hederagenin (HDG), excluding non-specific binding as cause for increase in netBRET ratio. BRET assays were performed in HEK293 cells transiently transfected with the Δ1-20-Nluc-NPFFR1-eYFP, data represent mean ± SEM of the netBRET ratio from n=3 experiments.

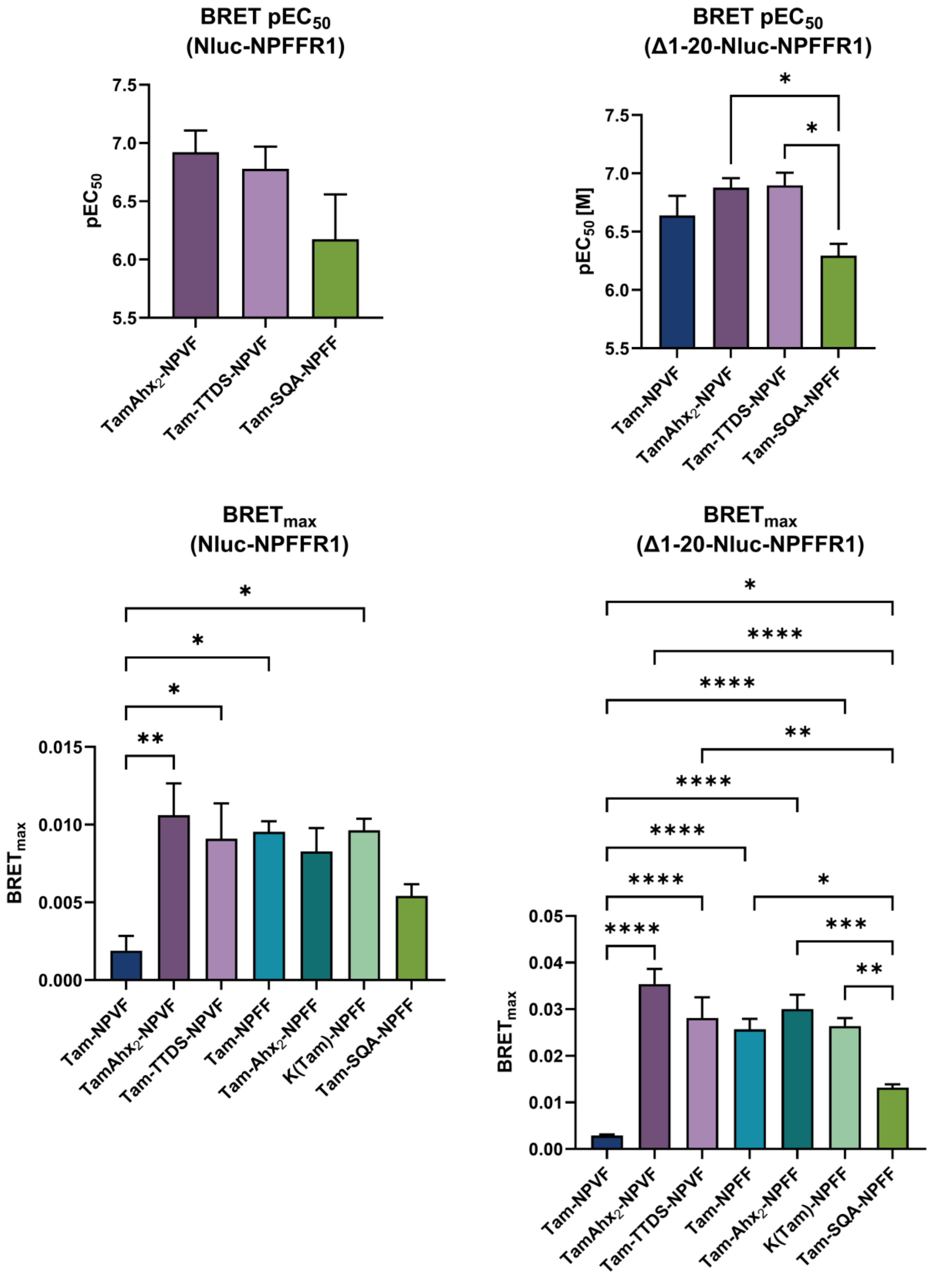

Figure S 2: Statistical analysis of pEC_{50} and $BRET_{max}$ values of the peptides at the Nluc-tagged full-length and truncated receptor. One-way ANOVA with multiple comparisons (Tukey) was performed. *, P<0.05; **, P<0.01; ***, P<0.001; and ****, P<0.0001.

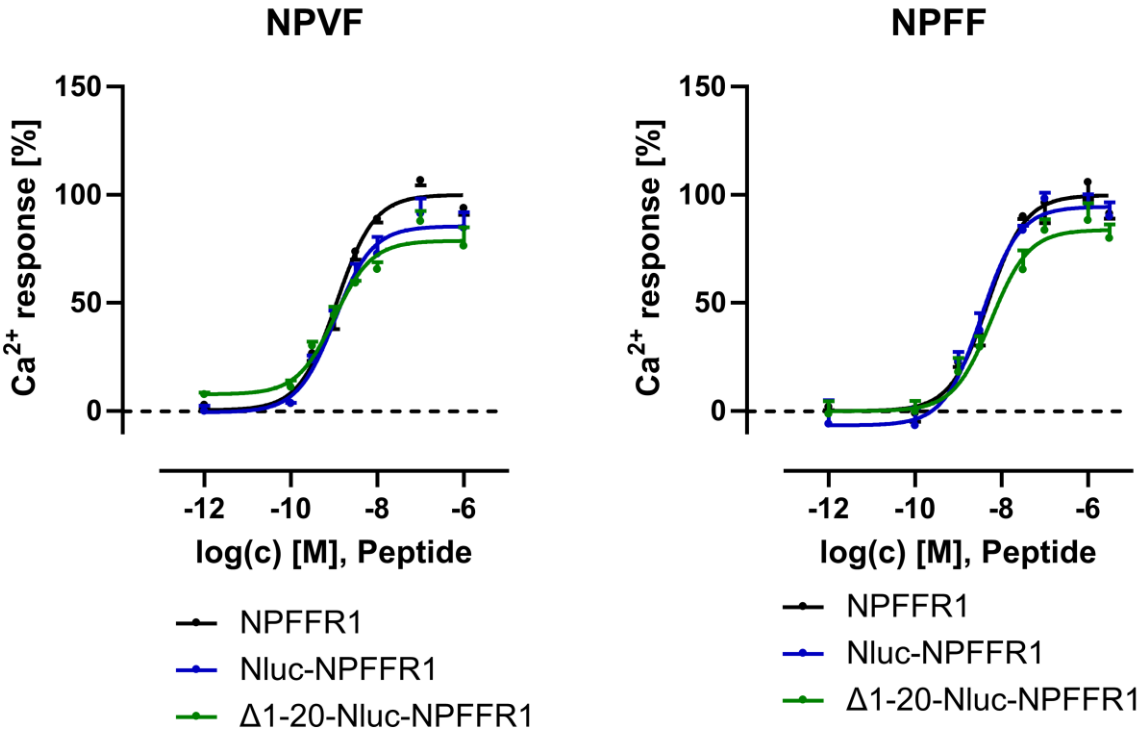

Figure S 3: Activity of NPVF and NPFF at the Nluc-labeled full-length and truncated NPFFR1 compared to the wildtype receptor. Ca^{2+} assays were performed in HEK293 cells transiently transfected with the NPFFR1, Nluc-NPFFR1 or Δ1-20-Nluc-NPFFR1 and the chimeric G protein $\Delta 6G\alpha_{qi4myr}$. Data represent mean ± SEM from n=3 experiments.
